# P-458. Genomic Characterization of Invasive and Colonizing Antibiotic-Resistant Enterobacterales in Infants

**DOI:** 10.1093/ofid/ofaf695.673

**Published:** 2026-01-11

**Authors:** Nabgha Farhat, Aspen Kremer, Alima Sajwani, Abigail Aron, Chao Qi, Cecilia Thompson, Joel Fisher, Egon A Ozer, Mehreen Arshad, Leena B Mithal

**Affiliations:** Lurie Children's Hospital, Chicago, IL; University of Chicago, Chicago, Illinois; Ann & Robert H. Lurie Children's Hospital, Chicago, Illinois; Anne & Robert H. Lurie Children’s Hospital of Chicago, Chicago, Illinois; Northwestern University Feinberg School of Medicine, Northwestern Memorial Hospital, Chicago, IL; Ann & Robert Lurie Children's Hospital, Chicago, Illinois; Northwestern Medicine Lake Forest Hospital, Chicago, Illinois; Northwestern University Feinberg School of Medicine, Chicago, Illinois; Lurie Children's/Northwestern, Chicago, Illinois; Ann and Robert H. Lurie Children's Hospital of Chicago, Chicago, Illinois

## Abstract

**Background:**

Infants are vulnerable to bacterial infections, including those caused by antibiotic-resistant Enterobacterales (AR-E). These bacteria can asymptomatically colonize the gut of young infants or cause invasive infections leading to morbidity and mortality. The objective of this study is to characterize differences between AR-E that cause invasive infections and those that are asymptomatic stool colonizers.

**Figure:**
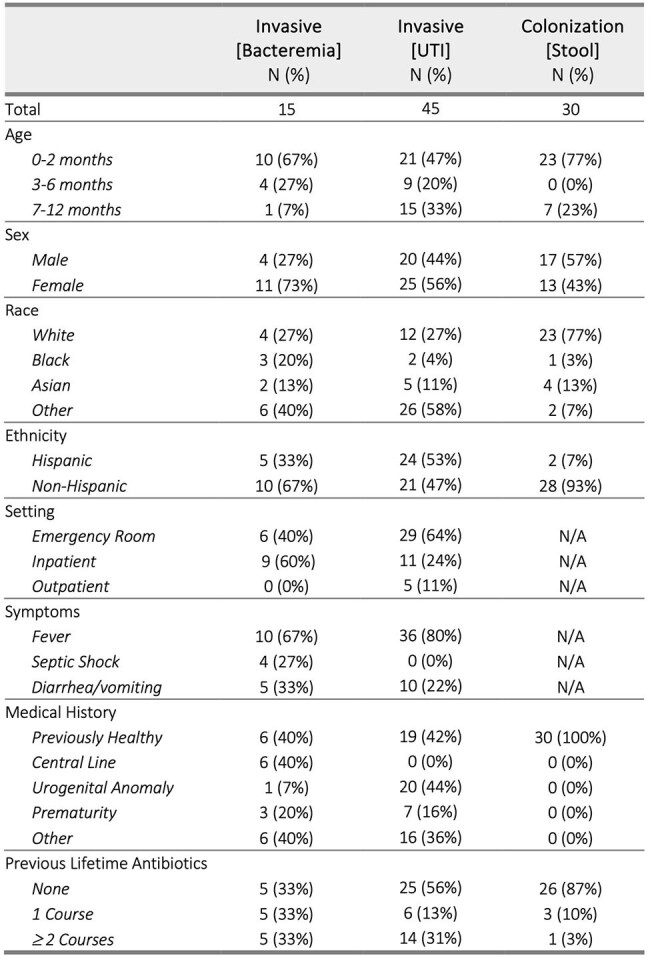
Clinical Characteristics of Patients with Antibiotic-Resistant Enterobacterales by Group

**Figure:**
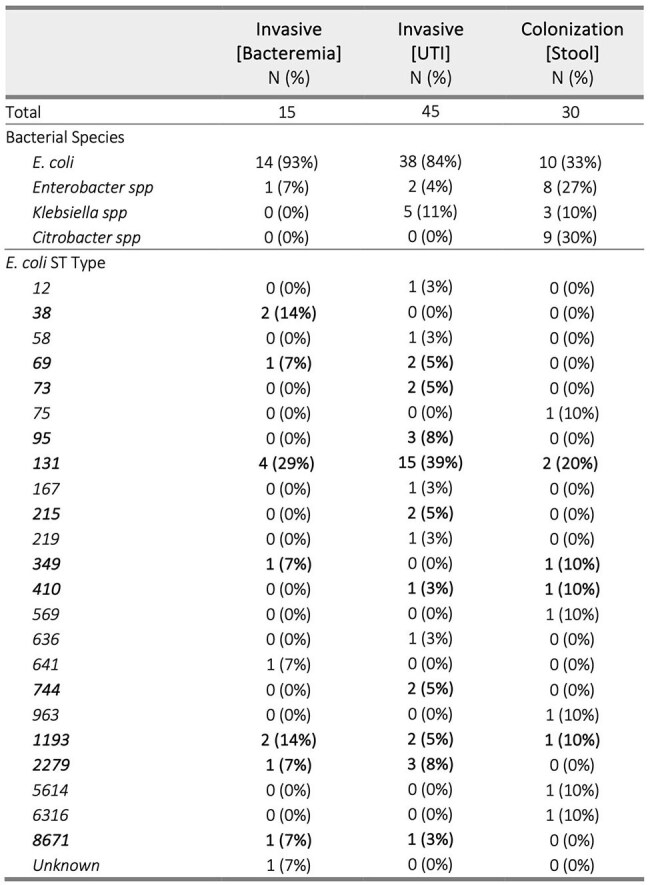
Microbiologic Characteristics of Antibiotic-Resistant Enterobacterales by Group Bolded sections represent E. coli ST Types that were observed > 2 times in this study

**Methods:**

AR-E isolates causing bacteremia and urinary tract infection (UTI) in infants less than 12 months of age over the past 10 years were retrieved from hospital clinical microbiology lab archives. Concurrently, AR-E were identified in stool of healthy community infants for an observational study titled “The Acquisition of Resistant Bacteria (ACQUIRE).” Invasive blood and urine isolates were compared to colonizing stool isolates.

Bacterial DNA was extracted then sequenced on the MiSeq Platform. De novo genome assembly was performed using SPAdes. Genomes were annotated using the RAST tool kit. A phylogenetic tree was constructed to visualize strain relatedness. Virulence genes were identified and compared between groups, with Benjamini-Hochberg correction for multiple comparisons.

**Figure:**
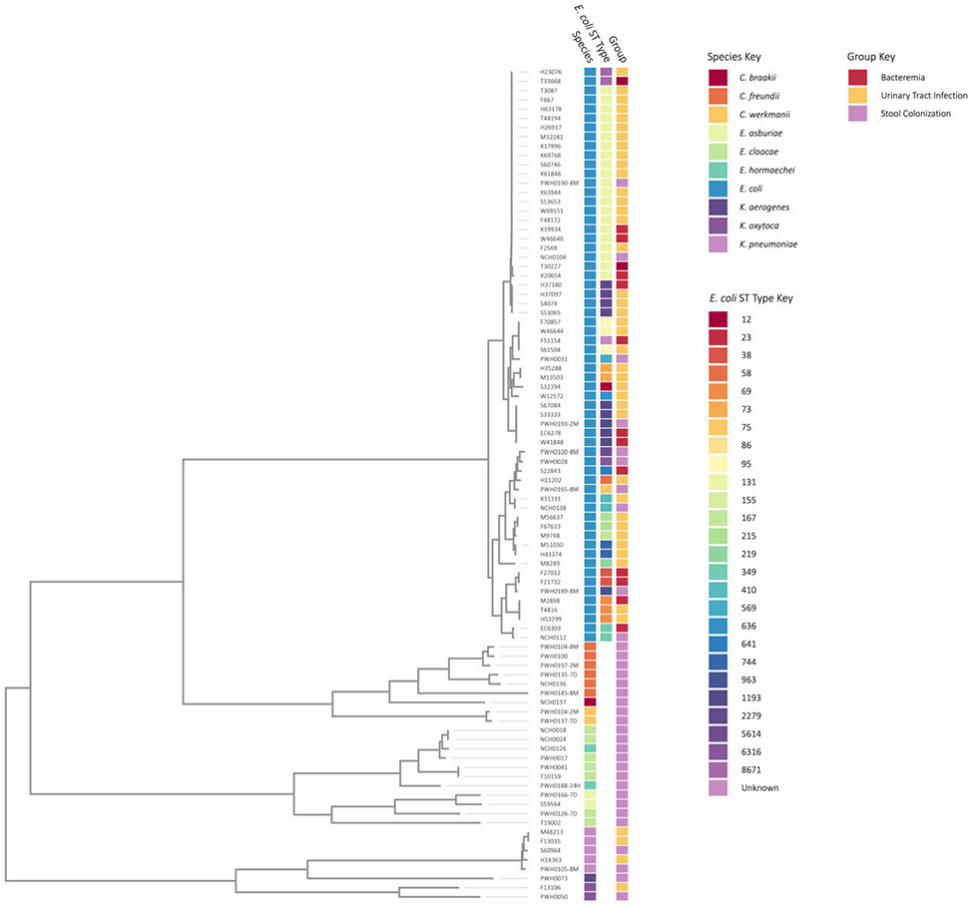
Phylogenetic Tree of AR-E Isolates Each label represents an AR-E isolate and the distance between isolates is a function of their relatedness. Clustering isolates with a more recent common ancestor are more closely related.

**Figure:**
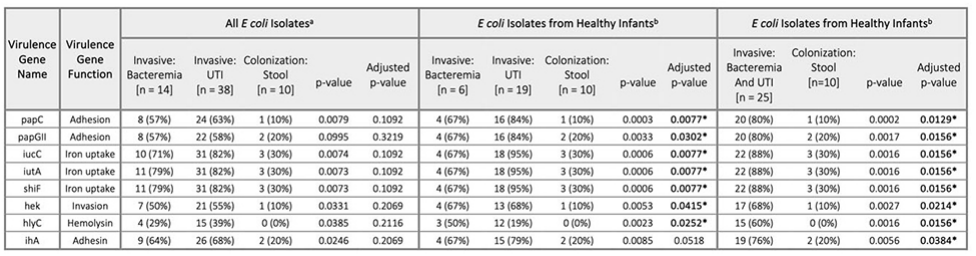
Virulence Gene Presence in E. coli Isolates by Group (a) All E. coli isolates that were identified in this study are included. (b) Only E. coli isolates from healthy infants are included, those without any medical history per chart review (e.g. no prematurity, no urogenital anomaly, no central line, etc.) *Adjusted p value < 0.05

**Results:**

We identified 15 cases of AR-E bacteremia, 45 cases of AR-E UTI, and 30 stool colonizing AR-E in infants. Characteristics of these patients and isolates are described in Table 1 and Table 2, respectively. A phylogenetic tree demonstrated clustering of invasive isolates as these were predominantly *E. coli*, most commonly strain ST131 (Figure 1). Eight virulence genes were significantly more frequent in *E. coli* isolates causing invasive infection rather than those asymptomatically colonizing healthy infants (Table 3).

**Conclusion:**

While many AR-E colonize the gut of healthy young infants, invasive infection was disproportionately caused by *E. coli*. The global pandemic *E. coli* strain ST131 was found to be both an asymptomatic colonizer and invasive pathogen. Compared to colonizing isolates, *E. coli* isolates causing invasive infection in healthy infants were more likely to have virulence genes implicated in bacterial adhesion, invasion, and iron uptake. These genes may inform future screening for virulent bacteria, or targeted interventions to reduce invasive infection.

**Disclosures:**

Cecilia Thompson, PhD, D(ABMM), bioMerieux: Honoraria|NaviDX Consulting: Consulting

